# A comparative study of machine learning models for predicting Aman rice yields in Bangladesh

**DOI:** 10.1016/j.heliyon.2024.e40764

**Published:** 2024-11-28

**Authors:** Taufiqul Islam, Tanmoy Mazumder, Md. Nishad Shahriair Roni, Md. Sadmin Nur

**Affiliations:** a Department of Urban and Regional Planning, Khulna University of Engineering & Technology (KUET), Khulna, 9203, Bangladesh; b Department of Computer Science and Engineering, Khulna University of Engineering & Technology (KUET), Khulna, 9203, Bangladesh

**Keywords:** Agricultural forecasting, Climate change, Random forest model, Food security, Data-driven insights, Sustainable development goals (SDGs)

## Abstract

Aman rice, a major staple crop in Bangladesh, is cultivated during the monsoon season and is highly dependent on climatic conditions such as rainfall and temperature. This study aims to identify the most effective machine learning models for predicting Aman rice yields by leveraging 52 years of historical data (1970–2022). Data preprocessing included outlier correction, statistical imputation, and aggregation of monthly averages for varibales like rainfall, temperature, humidity and others during the monsoon (June–September). Various machine learning models - Random Forest, Neural Network, Decision Tree, Linear Regression, and Gradient Boosting - were employed to capture yield trends under changing climatic conditions. Each model was evaluated based on Root Mean Squared Error (RMSE), R-squared (R^2^), and Mean Absolute Error (MAE). Random Forest emerged as the most accurate model showing robustness to climate variability through sensitivity analysis. While Gradient Boosting also performed well, though with slightly higher error margins. Linear Regression provided reasonable outputs, but it struggled with non-linear patterns. In contrast, Neural Networks and Decision Trees showed less accuracy in capturing intricate relationships between climate variables and rice yields. The Random Forest model predicts Aman rice yields to reach 133.31 metric tonnes by 2030 (34.11 % of total rice production) and 140 metric tonnes by 2050 (32.86 %). Climate projections suggest a rise in temperatures from 26.5°C to 37.41 °C in 2030 to 27.33°C-38.26 °C by 2050, with monsoon rainfall increasing slightly from 302.37 mm to 305.7 mm. These changes in climatic conditions could place additional stress on rice production, especially due to higher temperatures. The findings align with international studies highlighting the challenges that rising temperatures and fluctuating rainfall pose to crop yields. These findings emphasize the need for adaptive agricultural techniques and policies to mitigate climate change impacts on rice production, supporting food security and sustainable development in Bangladesh.

## Highlights

• This study evaluates multiple machine learning models to predict Aman rice yields in Bangladesh under varying climatic conditions.
• The Random Forest model demonstrated the highest accuracy, with sensitivity analysis validating its reliability across diverse climatic conditions, including variations in temperature and rainfall.
• The correlation between rice production and rainfall decreased from 0.5 (1970–1995) to −0.09 (1996–2022), reflecting reduced rainfall dependency.
• Emphasizes how temperature and rainfall affect the production of Aman rice.
• Highlights data-driven approaches for effective agricultural planning.

## 1 Introduction

Agriculture is a keystone of Bangladesh's economy, with rice being the most significant staple crop [1]. The nation's The country's three main rice crops - Boro, Aman, and Aus are seasonal varieties of Oryza sativa, cultivated in the winter, monsoon, and summer seasons, respectively [2]. Aman rice alone accounts for over 40 % of total production and nearly 50 % of cultivated rice land, relying heavily on monsoon rains. However, its dependence on rainfall and temperature makes it vulnerable to climate variability, with global climate change posing serious risks to rice production and food security. To address these challenges, machine learning supports yield forecasting and strengthens food security efforts amid changing weather patterns [3,4]. Over the past few decades, Bangladesh has experienced notable changes in weather patterns, leading to an increased frequency of extreme weather events such as floods, droughts, and cyclones. These climatic shifts have had a profound impact on agricultural productivity, particularly affecting the yield of Aman rice [5]. Historical trends in Aman rice production are closely tied to variations in rainfall and temperature. Understanding these trends is crucial for developing strategies to mitigate the adverse effects of climate change on agriculture [6].

Bangladesh is located in South Asia, a region notorious for significant rainfall during the monsoon season [7]. Approximately 20 %, 62.5 %, 15.5 %, and 2 % of Bangladesh's annual rainfall, which totals around 2,700 mm, occur during the pre-monsoon, monsoon, post-monsoon, and winter months, respectively [8]. Rice cultivation occupies over 78 % of Bangladesh's net farmed area. The country's food security is largely reliant on rice, as the 11.55 million hectares of cropland support the 169.04 million people living there, enabling self-sufficiency [9,10]. Notwithstanding the numerous initiatives to combat hunger, issues including escalating competition for natural resources, looming natural disasters and climate change, poverty, illiteracy, and illnesses are endangering food security and making the hunger crisis worse [11]. Reliable access for all individuals, at any given time, to adequate food required for an active and healthy life is called food security [12]. Bangladesh ranks 73rd out of 104 developing and transitioning nations in the most recent Global Hunger Index [13]. It is startling that, despite adequate food supply, 26 % of people remain chronically food insecure [14].

Three separate seasons can be distinguished in rice production: Boro (December/January–April), Aus (April/May–June/July), and Aman (July/August–November/December) [15]. The most common rice variety in Bangladesh, known as Aman, is rainfed during the monsoon season and is cultivated even in coastal regions. Aman is planted using two methods: direct seeding with Aus in March and April, and transplanting in July and August. Both varieties are harvested from November to December. However, late flooding can reduce the area planted with Aman, while summertime droughts can decrease the area for Aus cultivation [16]. In 2022, 394.94 Lac M. tonnes of rice were produced; of the total, 39.45 % came from Aman rice, which accounted for 154.26 Lac M. tonnes of output [17].

Given that the current production environment will change in the future due to decreasing cropland, labor resources, and increasing climatic sensitivity, the nation will need to enhance land productivity [10]. Several prior research works have compared machine learning algorithms for rainfall prediction, including the ARIMA model, Artificial Neural Network, Support Vector Machine (SVM), Self-Organizing Map (SOM), Multiple Linear Regression, and Lasso Regression [[18], [19], [20]]. Attributes like temperature, wind speed, and dew point were the considerations in terms of predicting rainfall with multiple regression models for Khartoum state [21]. Another study was designed by Liyew and Melese to evaluate the effectiveness of three machine learning methods—Extreme Gradient Boost, Random Forest, and Multivariate Linear Regression—using Mean Absolute Error and Root Mean Squared Error techniques, concluding that Extreme Gradient Boosting outperformed the others [22]. ANN (Artificial Neural Network) and MT (Model Tree) models were used for short-term rainfall prediction at Koyna Dam, Maharashtra, India, with both models showing equal performance in RMSE, Coefficient of Correlation, and Coefficient of Efficiency [23]. Six parameters (temperature, humidity, dew point, wind pressure, wind direction, and wind speed) were used to accurately forecast monthly and annual rainfall using LSTM and RNN models, yielding a 76 % accuracy rate [24]. Hong employed a hybrid model combining Support Vector Machines (SVM) and Recurrent Neural Networks (RNNs) with Chaotic Particle Swarm Optimization (CPSO) for rainfall forecasting in Taiwan, demonstrating significantly improved accuracy during typhoons, evidenced by lower normalized mean squared error (NMSE), higher coefficients of efficiency (CE), and coefficients of correlation (CC), making the RSVRCPSO model a viable option for capturing complex nonlinear rainfall patterns [25]. According to Shah et al., the experimentation demonstrates that ARIMA and Neural Network are the best for forecasting meteorological parameters, while the Random Forest model provides the best classification accuracy compared to other machine learning algorithms for next-season precipitation forecasting [26].

Machine learning models have been widely used in studies located in Bangladesh, India, and Pakistan to forecast rice harvest [[27], [28], [29], [30], [31], [32]]. Gandhi et al. used data from 27 districts in Maharashtra to demonstrate that machine learning techniques can estimate crop production in Indian regions cultivating rice under diverse climatic conditions, utilizing data from public government sources. Their calculations of mean absolute error (MAE), root mean square error (RMSE), relative absolute error (RAE), and root relative square error (RRSE) revealed that other methods outperformed SMO in crop yield prediction [33]. Bokhtiar et al. noted that, By 2050 and 2030, respectively, the ARIMA model projects a total rice crop demand of 42.6 million metric tonnes and 39.1 million metric tonnes. Two scenarios are included in the model: a business-as-usual scenario with a marginal surplus and a pessimistic scenario with a 3.62 million metric tons shortage [34]. Inyaem stated that Artificial Neural Network Technique (ANN) and Decision Tree Technique (DTT) are used to forecast the volume and selling price of rice produce for farmers. The model, tested on a dataset of farmer records using the CRISP-DM procedure, was assessed for performance using four options: Use Training Set, Cross-Validation with 10 Folds, Split 80-20, and Test Options [35]. Mo et al. developed an LSTM model to forecast rice yield using data collected over three years from 81 counties in Guangxi Province, China. The LSTM model, which outperformed standard recurrent neural networks in managing gradient dispersion and explosion, showed improved prediction accuracy when combined with meteorological parameters, as it was better at uncovering hidden information and fully utilizing data patterns [36].

An attempt was made by Bowden et al. to demonstrate how intricate non-linearities and interactions between the variability of rice output and the climate in India can be shown by random forest modeling [27]. Unfortunately, no similar literature exists in Bangladesh. The objective of this paper is to identify the most effective machine learning model for predicting Aman rice yields in Bangladesh to enhance agricultural decision-making and promote food security in the face of changing climate conditions. Firstly, we analyzed historical trends in Aman rice production, rainfall, and temperature from 1970 to 2022 and used various machine learning models to forecast future yields for 2030 and 2050, identifying the most accurate model for predictive purposes. Secondly, we provided comprehensive data-driven insights to support the formulation of agricultural policies and sustainable practices, promoting food security in Bangladesh and aligning with Sustainable Development Goal 12 by addressing the impacts of changing climate conditions. While several studies have applied machine learning models to forecast crop yields in regions like India, China, and other parts of the world, research specifically focusing on Aman rice in the context of Bangladesh's unique climatic and agronomic conditions is limited. This study bridges this gap by leveraging 52 years of historical data combined with future climate projections to develop predictive models tailored to the local context. Unlike previous works that focus primarily on general agricultural forecasting or rainfall prediction, our study provides a comprehensive comparison of multiple machine learning models to identify the most suitable approach for forecasting Aman rice yields under changing climate conditions. This work not only evaluates model performance but also offers insights into the sensitivity of each model to key climatic factors, providing actionable knowledge for policy development and climate adaptation strategies in Bangladesh.

## 2 Materials and methods

### 2.1 Study area

**Fig. 1:**
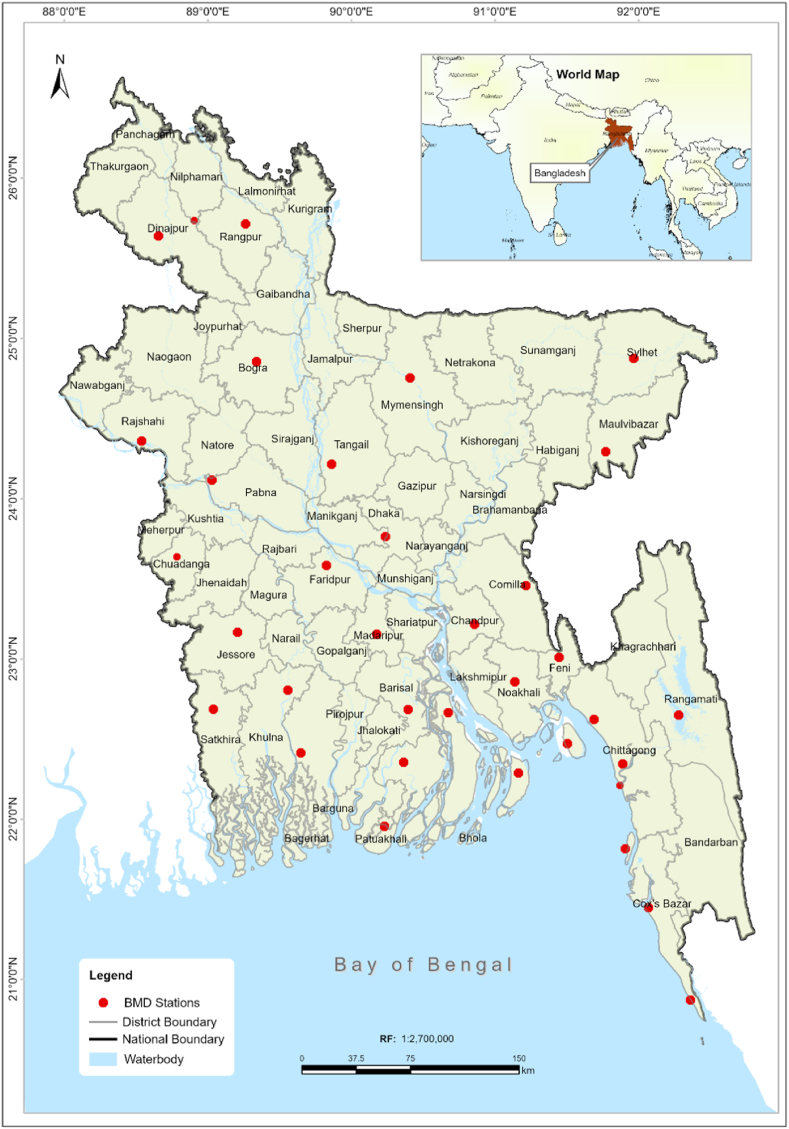
Study area.

The study area of this research covers the entire country of Bangladesh, a region significantly impacted by monsoon rains, which are crucial for predicting Aman rice [37]. Bangladesh has an agrarian economy in which rice is the dominant crop [16]. Geographically, it is a narrow, level plain bordered by the high Tibetan Plateau and the Himalayas to the north and the Bay of Bengal to the south [7]. The strategic placement of Bangladesh Meteorological Department (BMD) stations across the country provides critical data for this study, as depicted in Fig. 1.

### 2.2 Data collection

All data were gathered from the Bangladesh Meteorological Department (BMD), which operates thirty-five weather observation stations across the country. The study utilized data collected over 52 years, from June 1970 to September 2022. To forecast Aman rice yields, future climate projections were sourced from the World Bank Climate Change Knowledge Portal. Details on each of these factors, including their data sources and units are presented in Table 1.

**Table 1:** Factors with data sources and description, units, and their types.

Factors	Data Types	Data Source and Description	Unit
Rainfall	Historical Data	Bangladesh Meteorological Department (BMD), each of 35 meteorological stations, from 1970 to 2022, 52-year long period of data.	mm
Maximum Temperature			Degree Celsius
Minimum Temperature			Degree Celsius
Cloud Coverage			octs
Humidity			Percent
Sunshine			Hours
Wind Speed			Meter per second
Aman Rice Yields		Bangladesh Bureau of Statistics (BBS), from 1970 to 2022, 52-year long period of data.	Lac M. ton
Rainfall	Future Predicted Data	World Bank Climate Change Knowledge Portal	mm
Maximum Temperature			Degree Celsius
Minimum Temperature			Degree Celsius

### 2.3 Data processing

To ensure uniformity and accuracy in analysis, the raw data underwent a series of preprocessing steps. The initial data was collected and stored in CSV format, organized by weather station rather than district due to the absence of weather stations in some districts. The data covered 52 years from June 1970 to September 2022, focusing on variables such as rainfall, temperature, cloud coverage, humidity, sunshine, and wind speed.

Missing data points and outliers were identified and addressed to maintain the integrity of the dataset. Outliers were removed or corrected, and missing values were imputed using appropriate statistical methods to ensure a complete dataset for analysis. The data was aggregated monthly, with June, July, August, and September designated as the monsoon season. The mean values for these months were calculated to determine the average monsoon rainfall and other climatic factors. This approach provided a clear view of the monsoon's impact on Aman rice yields. Mean Monsoon Rainfall (MMR) was calculated (Equation (1)) as follows:

(1) $$Mean\;Mansoon\;Rainfall=\frac{Sum\;of\;JJAS\;\left(June,July,August,September\right)\;average\;rainfall}{4}$$

The projections, including variables such as temperature and rainfall, were sourced from the World Bank Climate Change Knowledge Portal, which provides data based on various climate models and greenhouse gas emission scenarios. To estimate conditions for the years 2030 and 2050, the mean values of projected climate data from 2020 to 2040 were used for 2030, and averages from 2040 to 2060 were used for 2050. This method offered a more stable representation of future climatic trends by smoothing out annual fluctuations and capturing broader changes over time. This approach ensured a comprehensive outlook on potential future climate conditions, minimizing the influence of short-term anomalies and providing a more reliable basis for assessing the impacts of climate change on Aman rice yields. The use of averaged projections allowed for more accurate predictions, supporting agricultural planning and climate adaptation strategies in Bangladesh. To facilitate comparison and analysis, all data, including both historical and projected values, were normalized using the Max-Min approach [38]. This method scales the data between 0 and 1, making it easier to handle and interpret in subsequent analyses. The normalization formula (Equation (2)) is as follows:

(2) $$y=\frac{\left(x-\min\left(x\right)\right)}{\left(\max\left(x\right)-\min\left(x\right)\right)}$$

where, x = Original value of a parameter; min(x) = Lowest value in the dataset x, max(x) = Highest value in the dataset x; and y = normalized value of x. The normalized data was stored in a new CSV file to ensure precision and effectiveness in the analysis phases.

### 2.4 Factor selection

Identifying the most predictive factors for the machine learning models was crucial. The correlation analysis was computed to evaluate the strength and direction of relationships between dependent and independent variables. Factors with high correlation coefficients (close to 1 or -1) and significant p-values were considered strong predictors. To further refine the selection, the Variance Inflation Factor (VIF) was calculated to assess multicollinearity among predictors. Factors with VIF values greater than 10 were considered to have high multicollinearity and were excluded from the models.

Factors with negative correlations and P-values greater than 0.05 were removed from the analysis. To enhance model accuracy and reduce redundancy, factors like wind speed, humidity, sunshine, and cloud coverage, which showed insignificant or negative correlations, were excluded from the final machine-learning models (Table 2). All Variance Inflation Factor (VIF) values remained relevant as they were below 10, streamlining the factor selection process and ensuring that only the most predictive variables were included in the analysis.

**Table 2:** Factors, correlation coefficients, P-values and VIF values.

Factors	Correlation Coefficients	P-Value	VIF
Aman Rice Production	1	0.0000	
Minimum Temperature	0.796439	0.0000	4.78
Maximum Temperature	0.88852	0.0000	6.04
Rainfall	0.456351	0.0006	4.19
Cloud Coverage	0.235173	0.0901	3.14
Humidity	−0.372265	0.0063	3.37
Sunshine	−0.247387	0.0743	5.02
Wind Speed	−0.092124	0.5120	1.61

### 2.5 Model development

To predict Aman rice yields, five machine learning models were selected: Random Forest, Gradient Boosting, Linear Regression, Decision Tree, and Neural Network. Each model was chosen for its distinct strengths in handling data patterns and relationships, ensuring a comprehensive comparison. Additionally, a sensitivity analysis was applied to understand how small changes in key input variables—maximum temperature, minimum temperature, and total rainfall—impact the performance of these models. This step ensured the robustness of the predictions, especially under variable climatic conditions [[39], [40], [41]].

Following the selection of independent variables, the dataset was split into 80 % for training and 20 % for testing. The training data was used to build and fine-tune the five machine learning models, while the testing data evaluated their predictive performance. Additionally, future climate projections from the World Bank Climate Change Knowledge Portal were also incorporated to enhance the models' forecasting capabilities. Each model was trained on the designated training dataset and tested for accuracy using multiple performance metrics. These metrics were then weighted and combined to calculate a Composite Score for each model. This score provided a comprehensive assessment of each model's predictive power. Based on their composite scores, the models were ranked, offering valuable insights into which models performed best for predicting Aman rice yields under both historical and future climate conditions. The details of each of the five models are provided below:

i) **Random Forest:** Since its introduction by L. Breiman in 2001, the random forest algorithm has shown to be an incredibly effective technique for both general-purpose classification and regression. The method performs exceptionally well in scenarios where there are many more variables than observations since it combines multiple randomized decision trees and averages their predictions. It also yields measures of varied relevance and is adaptable enough to be used on a variety of ad hoc learning tasks in addition to being large-scale problem-solving [42]. In China, South Korea, and Maharashtra State in India, the Random Forest algorithm is used to forecast agricultural yield, particularly rice [[43], [44], [45], [46]].
ii) **Artificial Neural Network:** The information-processing paradigm known as an Artificial Neural Network (ANN) is modeled after the way biological nervous systems, like the brain, handle information. A neural network (ANN) is made up of several layers of basic processing units known as neurons. The two tasks that a neuron completes are gathering inputs and producing an output. The theory, learning guidelines, and applications of the most significant neural network models, definitions, and computational styles are summarized through the use of ANN. A network's mathematical model clarifies the meaning of terms like inputs, weights, summing functions, activation functions, and outputs. Next, ANN assists in selecting the kind of learning for weight adjustments in response to parameter changes. The final steps in system analysis are ANN implementation, ANN training, and prediction quality [47].
iii) **Decision Tree:** One of the most popular approaches for representing classifiers in data classification is the use of decision tree classifiers. Researchers from a variety of disciplines and experiences have thought about the issue of expanding a decision tree using the data that is already available, including statistics, machine learning, and pattern recognition. Decision tree classifiers have been suggested for usage in many different disciplines, including medical disease analysis, text categorization, user smartphone classification, pictures, and many more [48].
iv) **Gradient Boosting:** By modifying ideas from classification theory, gradient boosting, a strong framework for predictor effect selection and estimation in a variety of regression models, originates from the statistical learning discipline [49]. Gradient Boosting (GB) is an iterative approach that generates a highly accurate prediction rule by combining simple parameterized functions with “poor” performance (high prediction error). Compared to other statistical learning techniques (such as neural networks and support vector machines) that often yield similar accuracy, GB produces findings that are easy to understand and requires minimal data preprocessing and parameter modification. The technique can be used for regression or classification problems from a range of response distributions (Gaussian, Bernoulli, Poisson, and Laplace), and it is quite resilient to less-than-clean data. Simple models are used to represent complex interactions, feature selection is carried out as a crucial step in the process, and missing values in the predictors are handled nearly without sacrificing information. Because of these qualities, GB is a strong insurance candidate for loss cost modelling [50].
v) **Linear Regression:** One method of supervised learning is regression. It can be applied to prediction and continuous variable modeling. Several applications of the linear regression technique include real estate price prediction, sales forecasting, exam score prediction for students, and stock exchange price movement forecasting. Regression is a supervised learning technique in which the output variable value is dictated by the values of the input variable. It uses labeled datasets. The simplest type of regression is linear regression, which is possible when the variables in the dataset have a linear relationship and involves fitting a straight line, or straight hyperplane, to the dataset [51].

### 2.6 Accuracy assessment

In the accuracy assessment section, the performance of the five models was evaluated using various metrics. Three key metrics were employed: Root Mean Squared Error (RMSE) (Equation (3)), R-squared (R^2^) (Equation (4)), and Mean Absolute Error (MAE) (Equation (5)). These metrics are calculated by the following equations:

(3) $$\text{Root}\;\text{Mean}\;\text{Squared}\;\text{Error}\;\left(\text{RMSE}\right):\sqrt{\frac{\sum {\left(Y_{i}-Y_{p}\right)}^{2}}{n}}$$

where, Y_i_ = Actual Value; Y_p_ = Predicted Value; n = No. of observations;

(4) $$R-\text{squared}\;\left(R2\right)\;\text{Score}:R^{2}=1-\frac{RSS}{TSS}$$

where, R^2^= Coefficient of Determination; RSS= Sum of squares of residuals; TSS = Total Sum of squares;

(5) $$\text{Mean}\;\text{Absolute}\;\text{Error}\;\left(\text{MAE}\right):\frac{\left|\left(Y_{i}-Y_{p}\right)\right|}{n}$$

where, Y_i_ = Actual Value; Y_p_ = Predicted Value; n = No. of observations;

A lower MAE indicates greater accuracy, a higher R^2^ score signifies a better fit, and a smaller RMSE suggests improved model performance. Based on these parameters, the Random Forest model performs the best, followed by Gradient Boosting and Linear Regression. In contrast, neural networks and decision trees show inadequate performance.

### 2.7 Selecting the optimal machine learning model

Since the model performs better when the RMSE and MAE values are lower, these two metrics' values were reciprocal. It was determined that a greater R^2^ value corresponds to a better match. To calculate the composite score, the total of these three metrics was divided by three. The model was ranked by composite score; a higher composite score indicated better model performance. The Random Forest model appears to be the most suitable for the study based on its higher composite score (Equation (6)) as indicated by Table 3. The equation is as follows:

(6) $$Composite\;Score=\frac{\left(\frac{1}{RMSE}+\frac{1}{MAE}+R^{2}\right)}{3}$$

**Table 3:** Model name, their rank, composite score with their accuracy assessment metrics.

**Rank**	**Model**	**Composite Score**	**RMSE**	**R**^**2**^	**MAE**
**1**	**Random Forest**	**0.46265**	**5.43995**	**0.96399**	**4.16462**
2	Gradient Boosting	0.42857	6.82479	0.94333	5.10614
3	Linear Regression	0.40653	7.45083	0.93245	6.53972
4	Decision Tree	0.37200	9.80808	0.88295	7.62818
5	Neural Network	−2.93810	89.91522	−8.83708	85.76825

### 2.8 Sensitivity analysis of the models

The sensitivity analysis revealed important insights into how each model responded to realistic climatic fluctuations. Key input variables—maximum temperature, minimum temperature, and total rainfall—were adjusted by ±10 % to evaluate the models' stability and predictive performance.

Among the models, Random Forest demonstrated the highest resilience, with only minor variations in RMSE and consistently strong R^2^ values. For instance, a 10 % increase in maximum temperature resulted in a reduced RMSE of 11.68 and an improved R^2^ of 0.834, indicating the model's robustness to temperature changes. Similarly, a 10 % decrease in total rainfall caused the RMSE to remain low at 11.18, while the R^2^ value stayed high at 0.848 [Table 4]. Gradient Boosting also performed well, although it exhibited greater sensitivity to feature changes. For example, a 10 % increase in minimum temperature led to an improved R^2^ of 0.868, suggesting better performance under those conditions. However, a 10 % decrease in maximum temperature caused the RMSE to rise to 13.07, highlighting its sensitivity to temperature fluctuations.

**Table 4:** Sensitivity analysis of machine learning models.

Model	Feature	Perturbation	RMSE	MAE	R2
Random Forest	Maximum Temperature	−10 %	12.29244	10.35363	0.81614
	Maximum Temperature	10 %	11.68415	8.66045	0.83389
	Minimum Temperature	−10 %	12.29603	10.02842	0.81604
	Minimum Temperature	10 %	10.91118	8.37343	0.85514
	Total Rainfall	−10 %	11.18022	8.50355	0.84791
	Total Rainfall	10 %	11.01110	8.16287	0.85248
Gradient Boosting	Maximum Temperature	−10 %	13.06935	10.84978	0.79217
	Maximum Temperature	10 %	11.36733	10.04216	0.84278
	Minimum Temperature	−10 %	12.24334	10.36107	0.81761
	Minimum Temperature	10 %	10.39803	9.45744	0.86845
	Total Rainfall	−10 %	10.81109	8.75481	0.85779
	Total Rainfall	10 %	10.38332	7.75704	0.86882
Linear Regression	Maximum Temperature	−10 %	11.42258	10.30487	0.84124
	Maximum Temperature	10 %	8.13379	6.30533	0.91950
	Minimum Temperature	−10 %	8.78997	7.58052	0.90599
	Minimum Temperature	10 %	8.98258	7.87899	0.90182
	Total Rainfall	−10 %	9.48991	8.44381	0.89042
	Total Rainfall	10 %	8.69930	7.02520	0.90792
Decision Tree	Maximum Temperature	−10 %	14.20196	11.82091	0.75459
	Maximum Temperature	10 %	12.00656	10.76455	0.82460
	Minimum Temperature	−10 %	13.34526	11.12545	0.78330
	Minimum Temperature	10 %	12.71758	10.57000	0.80321
	Total Rainfall	−10 %	12.34084	9.63273	0.81469
	Total Rainfall	10 %	12.39923	10.22727	0.81294
Neural Network	Maximum Temperature	−10 %	13.00722	11.25786	0.79414
	Maximum Temperature	10 %	11.94449	9.04258	0.82641
	Minimum Temperature	−10 %	12.54238	10.72795	0.80859
	Minimum Temperature	10 %	12.25050	9.57249	0.81740
	Total Rainfall	−10 %	12.42531	10.89750	0.81215
	Total Rainfall	10 %	12.65366	9.40299	0.80518

Linear Regression provided reliable predictions for linear patterns but struggled with non-linear relationships. In favorable conditions, such as a 10 % increase in total rainfall, the R^2^ improved to 0.908. However, the RMSE showed considerable variability, indicating the model's limitations in handling non-linear climatic changes. In contrast, the Decision Tree and Neural Network models displayed higher sensitivity to input variations, which impacted their accuracy. A 10 % decrease in maximum temperature led to a drop in the Decision Tree's R^2^ to 0.755, reflecting its inability to manage temperature changes effectively. The Neural Network model also struggled with minor input changes, as a 10 % decrease in total rainfall caused the RMSE to increase to 12.43, with the R^2^ remaining relatively low at 0.812, suggesting reduced reliability for complex datasets.

**Fig. 2:**
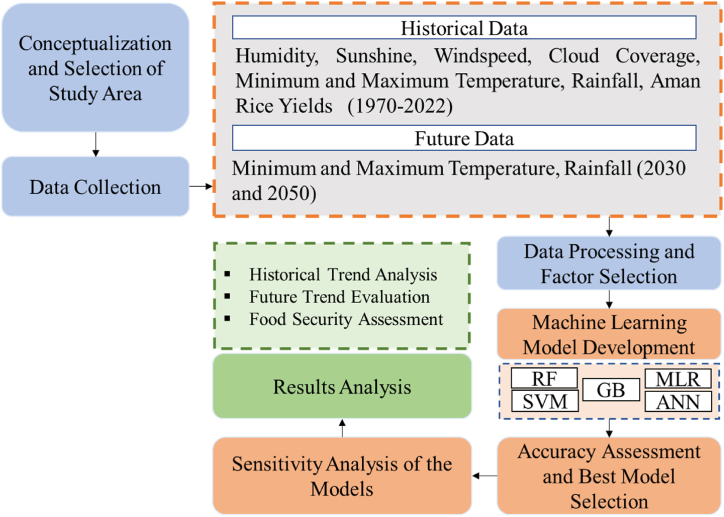
Flowchart on overall methodological process.

This analysis highlights Random Forest as the most robust model under varying climatic conditions, followed by Gradient Boosting. On the other hand, Linear Regression, Decision Tree, and Neural Network models showed varying degrees of sensitivity, making them less suitable for datasets with complex non-linear patterns. The methodology attempts to give a concise overview for choosing the optimal machine learning model to estimate Aman rice production, starting with data collection and continuing through detailed analysis and presentation. Fig. 2 shows the study's methodological framework.

## 3 Results

### 3.1 Current situation of Aman rice, monsoon rainfall, maximum and minimum temperature

**Fig. 3:**
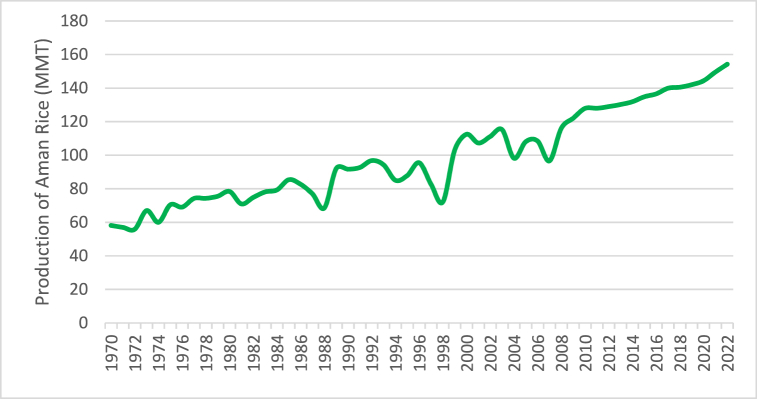
Current situation of Aman rice production.

Fig. 3 depicts the annual trend of Aman rice output from 1970 to 2022, which shows a range of changes over time but is generally increasing. Around 154.26 lac M. tonnes will be produced at its highest point in 2022, while approximately 55.87 lac M. tonnes will be produced at its lowest point in 1972. The trend line shows a consistent upward trend over time. The production values of 68.57, 72.17, 98.2, and 96.62 lac M. tonne for the years 1988, 1998, 2004, and 2007, respectively, indicate a significant decrease in comparison to the overall trend of Aman rice production. A substantial decrease in both cultivated area and yield was experienced by the production during the devastating floods that struck in 1988, 1998, 2004, and 2007 [52].

**Fig. 4:**
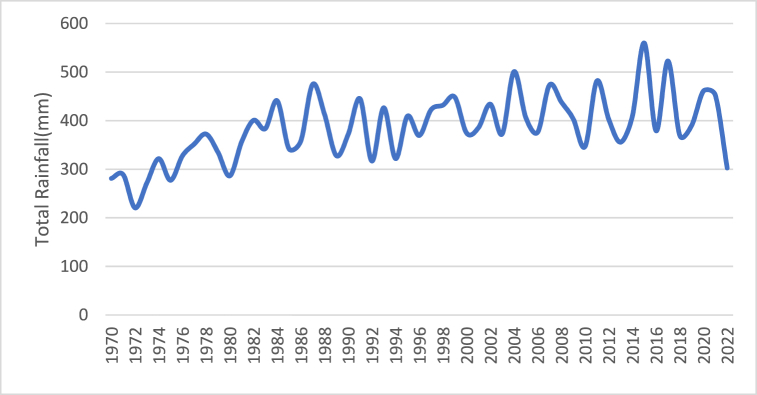
Current situation of monsoon rainfall.

Fig. 4 shows how the amount of rain received during the monsoon varies from 1970 to 2022. Apart from a few significant outlier years, the amount of rain is almost constant, with 2015 appearing to have had the highest amount at 559.74 mm.

**Fig. 5:**
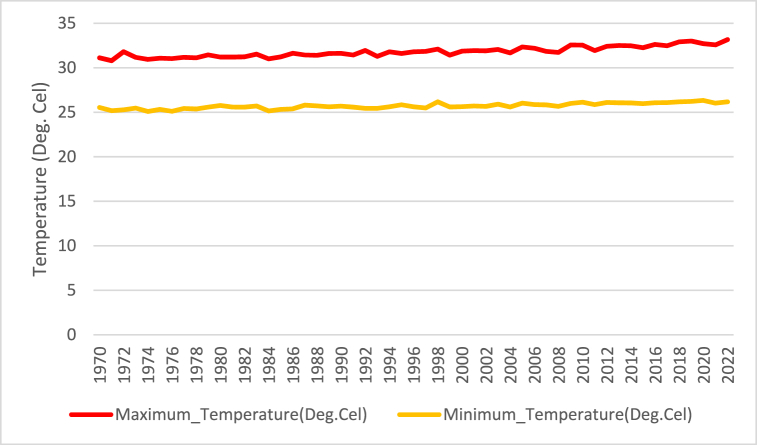
Current situation of maximum and minimum temperature.

Fig. 5 displays the changes in maximum and minimum temperatures from 1970 to 2022. With a few slight variations over 52 years, the maximum temperature trend is shown by the red line. In 2022, the highest recorded temperature is 33.17 °C. The trend of the lowest temperature is shown by the orange line. Like the highest temperature, it displays stability with just minor variations. In 1974, the lowest recorded temperature was roughly 25.09 °C. Without much variation, the temperature lines for the highest and minimum temperatures run parallel. This demonstrates a steady trend in temperature changes over the course of the 52 years.

### 3.2 Future situation of Aman rice, monsoon rainfall, maximum and minimum temperature

**Fig. 6:**
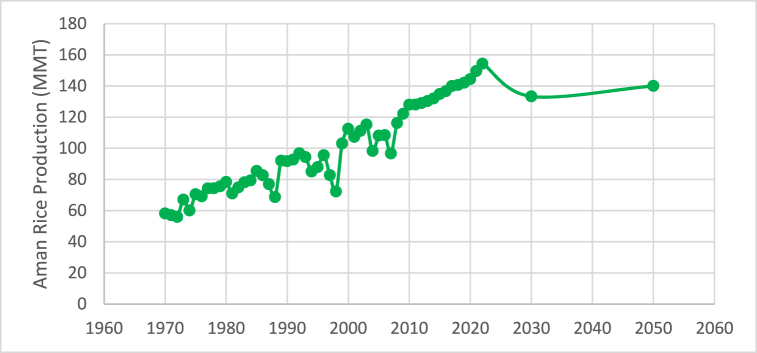
Future predicted situation of Aman rice production.

Aman rice production is estimated to reach 140 lac M tonnes in 2050 and 133.31 lac M tonnes in 2030 according to the Random Forest model, which is the best model (Fig. 6, Fig. 9).

**Fig. 7:**
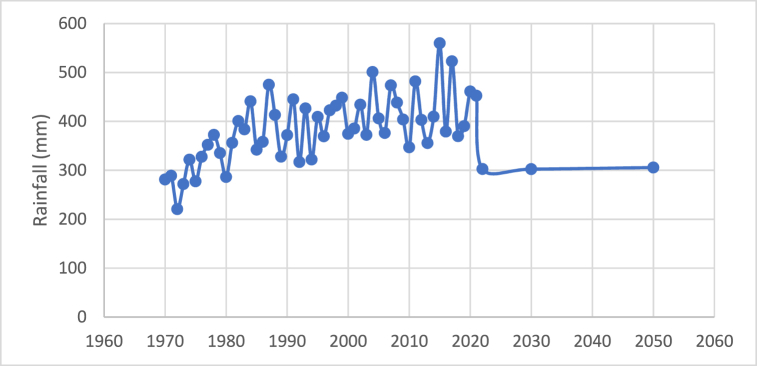
Future predicted situation of rainfall.

Fig. 7 predicts total rainfall of 302.37 mm in 2030 and 305.7 mm in 2050 from the World Bank Climate Change Knowledge Portal.

**Fig. 8:**
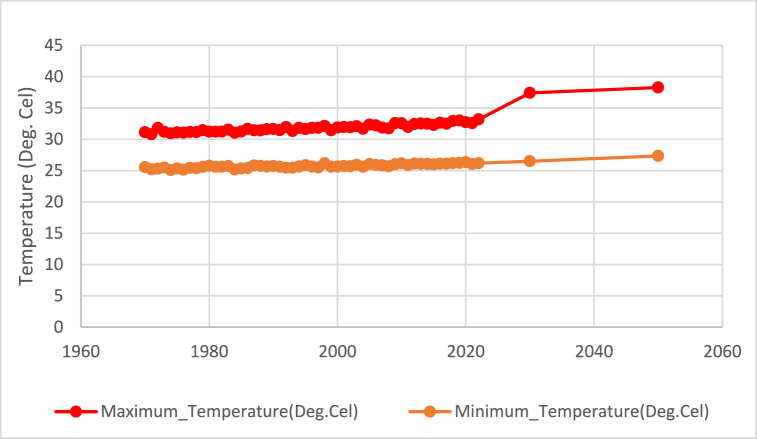
Future predicted situation of Maximum and Minimum temperature.

The estimated minimum and maximum temperatures for 2030 are 26.5 °C and 37.41 °C, respectively, while those for 2050 are 27.33 °C and 38.26 °C from World Bank Climate Change Knowledge Portal shown in Fig. 8. Both the minimum and maximum temperatures show a gradual increase, indicating a warming trend.

**Fig. 9:**
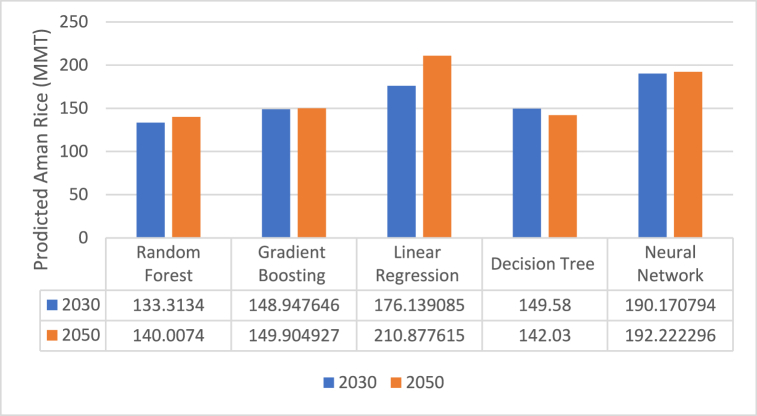
Comparison between different ML models.

### 3.3 Validation and comparison of model results

The model's predictions for Aman rice for the years 2030 and 2050 are displayed in the table, with each model's prediction demonstrating a discernible difference between the two years following Fig. 9. Among these, Random Forest shows the lowest amount for the year 2030—133.313 lac M tonnes—while Neural Network shows the maximum amount—190.17 lac M tonnes. The lowest value for 2050 is 140.0074 lac M tonnes, as shown by Random Forest, while the greatest value, 210.877 lac M tonnes, is indicated by Linear Regression. Across all categories, the model's projection for 2030 is consistently less than that for 2050.

### 3.4 Result analysis

When it came to predicting Aman rice yields, the Random Forest model performed admirably. The model predicts that by 2030, Aman rice production will make up roughly 34.11 % (133.31 metric tonnes) of Bangladesh's 391 metric tonnes of overall rice production. With an anticipated yield of 140 metric tonnes out of 426 metric tonnes, Aman rice's share is predicted to marginally decline to 32.86 % by 2050 [34]. Understanding how Aman rice will contribute to food security in the future as climate conditions change depends on these projections.

Significant changes in climate variables are predicted by the model. Temperatures are predicted to vary from a minimum of 26.5 °C to a maximum of 37.41 °C by 2030, with monsoon rainfall of approximately 302.37 mm. These numbers indicate a considerable rise in temperature relative to present norms, which may put rice harvests under stress. According to projections, monsoon rainfall would slightly increase to 305.7 mm by 2050, and temperatures will rise far more, from 27.33 to 38.26 °C. These changes in the climate are consistent with international research, such as that conducted by Gandhi et al. (2016) and Hong (2008), that highlights the growing difficulties that crop yields face due to rising temperatures and unpredictable rainfall [25,28].

The effects of climate variability on rice yields have been thoroughly examined in other regions, including China and India, and our results are in line with comparable studies carried out there. For example, Bowden et al. (2023) emphasized the application of Random Forest models to capture intricate relationships between Indian rice yields and climate variables [27]. In a similar vein, Mo et al. (2021) showed how well sophisticated machine-learning models forecast agricultural yields in China under shifting meteorological conditions [36]. Our study's comparison of these estimates highlights the necessity of adaptable agricultural techniques in Bangladesh to reduce the hazards brought on by shifting monsoon patterns and warming temperatures.

## 4 Discussion

This study highlights the effectiveness of machine learning models in forecasting Aman rice yields in Bangladesh, emphasizing the impact of climate factors like rainfall and temperature. The Random Forest model emerged as the most reliable tool for predicting Aman rice yields under varying climatic conditions. Its ability to handle non-linear relationships between variables ensured the lowest RMSE and the highest R^2^ score among the models evaluated. Similar conclusions were reported by Bowden et al. (2023), where Random Forest was successfully used to predict rice production in India amidst monsoon variability [27]. This model's superior performance underscores its adaptability to complex datasets, making it particularly relevant for regions like Bangladesh, where climate variability plays a significant role. The sensitivity analysis revealed that the Random Forest model maintained its predictive power despite changes in key climatic variables, outperforming Gradient Boosting, Linear Regression, and Neural Networks. This finding is consistent with studies by Mo et al. (2021) and Shah et al. (2018), which highlighted the Random Forest model's robustness in agricultural forecasting across different climatic regions [26,36]. The ability to maintain accuracy under varying conditions suggests the model's suitability for policy planning in Bangladesh's climate-affected agricultural sector.

The study found a declining dependency of Aman rice production on monsoon rainfall, with the correlation coefficient dropping from 0.5 in 1970–1995 to −0.09 in 1996–2022. This trend aligns with observations by Mainuddin et al. (2015), who highlighted the growing influence of irrigation systems over rainfall in sustaining crop production in Bangladesh [55]. It suggests that technological advancements such as improved irrigation have mitigated the impact of fluctuating rainfall on crop yields, though future unpredictability of rainfall necessitates ongoing monitoring. The study projects a temperature increase from 26.5°C to 37.41 °C in 2030 to 27.33°C-38.26 °C by 2050, potentially stressing Aman rice yields. Similar trends have been reported by Gandhi et al. and Hong, who emphasized that rising temperatures negatively affect crop yields by disrupting growth cycles and increasing evapotranspiration [25,28,33]. Thus, future agricultural policies in Bangladesh must account for temperature stress and incorporate adaptive practices, such as drought-tolerant rice varieties, to maintain yield levels.

The forecasted yields for Aman rice—133.31 metric tonnes in 2030 and 140 metric tonnes in 2050—indicate a steady increase, albeit with a slightly declining share in total rice production. These projections align with national food security strategies, as outlined by Kabir et al. (2021), which emphasize the need for adaptive agricultural policies to meet future demands [10]. In line with global studies, the use of machine learning models can support timely policy interventions, resource allocation, and the development of climate-resilient agricultural practices. Bangladesh, the eighth most populous nation in the world and the most South Asian nation, is overpopulated. By 2050, the population is expected to reach 340.3 million and 236.3 million, respectively, based on logistic and exponential growth models [53]. According to another analysis, Bangladesh's population is expected to reach 215.4 million in 2050 from 186.0 million in 2030 [54]. Bangladesh has recently become self-sufficient in rice, mostly as a result of higher yields and a significant expansion in the area under groundwater irrigation for dry season rice over the previous few decades [55]. According to projections, the overall crop demand for rice will be 42.6 MMT in 2050 and 39.1 MMT in 2030. The pessimistic scenario estimated that climatic shocks might cause a shortfall of 3.62 MMTs in 2030 and 1.93 MMTs in 2050, whereas the business-as-usual scenario anticipated that there will be a marginal surplus of rice in 2030 and 2050 [34].

In Bangladesh, food security is synonymous with rice security, deeply rooted in both political and cultural traditions. For the past ten years, the nation has produced an excess of rice to feed almost 165 million people. This “self-sufficiency momentum” would need to continue to fulfil the rising demand brought on by an expanding future population [10]. The model predictions can be directly applied to policy decision-making in several ways. For example, the forecasts of Aman rice yields can guide resource allocation by identifying regions that may require additional support during low-yield years, such as targeted subsidies or improved irrigation infrastructure. The models can also be used to issue timely advisories to farmers, helping them adjust planting schedules or adopt appropriate crop varieties in response to predicted climate conditions. Furthermore, these predictions can inform long-term governmental planning, such as designing climate adaptation strategies and allocating resources for agricultural research and development. By integrating model outcomes into policy frameworks, decision-makers can enhance food security and resilience against climate variability. The findings can also inform comparative research in other South Asian countries, such as India and Pakistan, where monsoon-dependent rice agriculture shares similar climatic and agricultural conditions. Evaluating the performance of machine learning models like Random Forest in these regions, with their varying rainfall patterns, temperature trends, and agricultural practices, could provide valuable insights into the robustness and adaptability of such models.

The study's limitations include the insufficient number of BMD stations, along with missing data and outliers, which reduced the accuracy of the models. Additionally, the study primarily relies on historical weather data and projections from climate models, which are inherently uncertain and subject to variability, particularly for long-term forecasts. Furthermore, the study focuses solely on climatic factors, excluding other socioeconomic variables like market dynamics, government policies, or farming practices, which also influence agricultural productivity [56]. Incorporating these additional factors in future research would provide a more comprehensive understanding of the complexities affecting Aman rice production. Finally, while the Random Forest model performed well, exploring hybrid models or integrating more recent machine learning techniques could further enhance predictive accuracy.

Future research could benefit from incorporating more comprehensive climate data and examining regional climate variations, such as droughts or unseasonal rainfall, to better understand their impact on rice production. Additionally, expanding the model to include socioeconomic factors, such as changes in farming practices and government interventions, would offer deeper insights into the effects of climate change on rice yields. These improvements would help in designing targeted agricultural policies that address the specific needs of each region. In China, for example, sustainable agriculture plays a vital role in achieving food security amidst climate variability. The agricultural sector faces challenges from unpredictable weather patterns that affect crop yields, underscoring the need for precise forecasting methods. Machine learning models have proven to be valuable tools in this context, enhancing the accuracy of forecasts and informing policy decisions by addressing the complex relationships between climate variables and agricultural productivity [57].

## 5 Conclusion

This study marks a significant advancement in the application of machine learning for forecasting Aman rice production. It underscores the importance of aligning agricultural practices with sustainable development goals (SDG 12), particularly by promoting responsible consumption and production [58]. The research demonstrates that sustainable agriculture practices, including precision agriculture, drought-resistant rice cultivars, and improved irrigation systems, are essential to balance economic growth and environmental sustainability through effective water management and the empowerment of small-scale farmers and service providers [59].

The Random Forest model emerged as the most robust tool for predicting Aman rice yields, offering the most reliable insights under varying climatic conditions. It demonstrated superior accuracy compared to other models such as Gradient Boosting, Linear Regression, Decision Tree, and Neural Networks. This model predicted that Aman rice production would reach 133.31 metric tonnes by 2030, contributing 34.11 % of total rice production, and 140 metric tonnes by 2050, accounting for 32.86 %. However, the findings indicate potential challenges, as rising temperatures and fluctuating rainfall could stress future rice yields.

Forecasting Aman rice production and meeting future demand will require substantial investments along with supportive government policies. Collaborative efforts between the public and private sectors are essential to provide the necessary financial resources for infrastructure development, technological adaptation, and agricultural research. Region-specific climate-smart technologies, such as precision agriculture, drought-resistant rice cultivars, and improved irrigation systems, are needed to enhance Aman rice output and align production with SDG 12 [37]. As climate variability threatens crop production, accurate forecasting becomes increasingly important to mitigate the adverse effects of changing weather patterns on food production. Machine learning models are proving to be valuable tools for forecasting yields, reducing the impact of climate change, and promoting sustainable agriculture [57,59,60].

Developing a strategic reserve of rice grains and establishing a reliable supply chain system could further enhance food security by minimizing the risks associated with potential shortages. This research provides data-driven insights that can guide policy formulation, resource allocation, and regulatory frameworks to secure food availability. The research aligns with global efforts in agricultural forecasting by comparing its findings with those from studies conducted in India, China, and parts of Africa [27,39,41,61,62]. For instance, Bowden et al. (2023) demonstrated how Random Forest models can effectively manage complex monsoon variability and rice production in India [27]. The Random Forest model's robust performance in predicting Aman rice yields under varying climatic conditions is consistent with outcomes in other regions, reinforcing the global relevance of data-driven approaches in agriculture [56,63]. These insights expand the applicability of the study beyond Bangladesh, providing valuable perspectives for shaping agricultural policies in other monsoon-dependent regions prone to climate variability. In addition to enhancing national agricultural planning, this study provides a framework that can be applied across South Asia and Africa, where fluctuating climates pose growing threats to food security.

In conclusion, this research establishes a solid foundation for developing evidence-based policies, resource allocation strategies, and regulatory frameworks that support sustainable rice production. The study not only contributes to Bangladesh's agricultural policy but also aligns with global efforts to improve forecasting under changing climatic conditions. These findings provide a basis for international comparisons, especially in monsoon-dependent agricultural systems, encouraging future research collaborations in regions facing similar challenges.

## CRediT authorship contribution statement

**Taufiqul Islam:** Writing – review & editing, Writing – original draft, Visualization, Validation, Software, Methodology, Investigation, Formal analysis, Data curation, Conceptualization. **Tanmoy Mazumder:** Writing – review & editing, Visualization, Validation, Supervision, Resources, Methodology, Investigation, Conceptualization. **Md. Nishad Shahriair Roni:** Writing – review & editing, Validation, Formal analysis, Data curation. **Md. Sadmin Nur:** Writing – review & editing, Visualization, Formal analysis.

## Data availability statement

The datasets generated during and/or analyzed during the current study are available from the corresponding author on reasonable request.

## Ethical approval

The authors declare that the research article is compliance with the ethical standards of this journal.

## Funding statement

This research did not receive any specific grant from funding agencies in the public, commercial, or not-for-profit sectors.

## Declaration of competing interest

The authors declare that they have no known competing financial interests or personal relationships that could have appeared to influence the work reported in this paper.
